# Successful elimination of non-neural cells and unachievable elimination of glial cells by means of commonly used cell culture manipulations during differentiation of GFAP and SOX2 positive neural progenitors (NHA) to neuronal cells

**DOI:** 10.1186/1472-6750-8-56

**Published:** 2008-07-19

**Authors:** Monika Witusik, Sylwester Piaskowski, Krystyna Hulas-Bigoszewska, Magdalena Zakrzewska, Sylwia M Gresner, S Ausim Azizi, Barbara Krynska, Pawel P Liberski, Piotr Rieske

**Affiliations:** 1 Medical University of Lodz, Department of Molecular Pathology and Neuropathology, 8/10 Czechoslowacka str. 92-216 Lodz, Poland; 2 Department of Neurology, Temple University School of Medicine, 3401 N Broad St. 558 Parkinson Pavilion, Philadelphia, PA 19140, USA

## Abstract

**Background:**

Although extensive research has been performed to control differentiation of neural stem cells – still, the response of those cells to diverse cell culture conditions often appears to be random and difficult to predict. To this end, we strived to obtain stabilized protocol of NHA cells differentiation – allowing for an increase in percentage yield of neuronal cells.

**Results:**

Uncommitted GFAP and SOX2 positive neural progenitors – so-called, Normal Human Astrocytes (NHA) were differentiated in different environmental conditions to: only neural cells consisted of neuronal [MAP2+, GFAP-] and glial [GFAP+, MAP2-] population, non-neural cells [CD44+, VIMENTIN+, FIBRONECTIN+, MAP2-, GFAP-, S100β-, SOX2-], or mixture of neural and non-neural cells.

In spite of successfully increasing the percentage yield of glial and neuronal *vs*. non-neural cells by means of environmental changes, we were not able to increase significantly the percentage of neuronal (GABA-ergic and catecholaminergic) over glial cells under several different cell culture testing conditions. Supplementing serum-free medium with several growth factors (SHH, bFGF, GDNF) did not radically change the ratio between neuronal and glial cells – i.e., 1,1:1 in medium without growth factors and 1,4:1 in medium with GDNF, respectively.

**Conclusion:**

We suggest that biotechnologists attempting to enrich *in vitro *neural cell cultures in one type of cells – such as that required for transplantology purposes, should consider the strong limiting influence of intrinsic factors upon extracellular factors commonly tested in cell culture conditions.

## Background

So-called NHA (Normal Human Astrocytes) cells belong to the class of GFAP-positive neural progenitors. Expression of neuronal and glial markers during differentiation of these cells is regulated in accordance with the "model of discordant phenotypes suppression" [1-3]. This model states that before differentiation, markers belonging to diverse lineages are expressed by stem cells or progenitors; whereas during differentiation, genes superfluous in derived lines are silenced. In accordance with this concept, uncommitted neural progenitors (NHA) co-express glial [GFAP, CD44], neuronal [β-III-TUBULIN, MAP2] and progenitor [NESTIN] markers. GFAP positive neural progenitors (NHA) derivatives differentiating to the neuronal lineage demonstrated silencing of glial and progenitor markers [GFAP, CD44, NESTIN] expression, while those differentiating to the glial lineage showed silencing of neuronal and progenitor markers [β-III-TUBULIN, MAP2, NESTIN] expression. However expression of MAP2 and β-III-TUBULIN is definitely increased in neuronal derivatives of NHA [1,2].

We could not prove so far that the coexpression of glial and neuronal markers is a consequence of physiological process. To this end we consider our investigation as biotechnologically useful, and we do not imply that a similar scenario has to occur *in vivo*. However report revealing presence of neural stem cells or progenitors expressing neuronal markers *in vivo *has been published lately by Walker et al [4]. Coexpression of glial and neuronal markers in neural progenitors was also presented [5]. Moreover, article showing human fetal astrocytes coexpressing *in vivo *GFAP, β-III-TUBULIN and MAP2 was published [6]. The expression of β-III-TUBULIN in conjunction with MAP2 in GFAP-positive radial glia has been considered as suggestion of neuronal-glial bipotentiality [7]. Radial glia are recognized as cells presenting NSCs properties [7,8].

According to the suppression of discordant phenotypes model, the expression of markers characteristic for particular lineages in progenitor cells allows for the presumption of potential derivatives that can be obtained after differentiation. This model, however, is of very limited help in the biotechnological regulation of differentiation. Hence, we took into account others such as the instructive, stochastic, and continuum models – considering the possibility that they can help to increase the percentage yield of a given required cell type via cell culture manipulations.

The instructive (deterministic) model recognizes growth factors as elements which determine the fate of stem cells – thereby, triggering a particular differentiation pathway [9,10]. Extensive research has shown that the fate of stem cells can be influenced by exogenous factors. However, their response to environmental signals often emerge to be random and difficult to predict. To this end, biologists have realised that an alternative model to the deterministic one would be required to explain better how the differentiation process is regulated. Hence, difficulties in predicting the response of stem cells have inspired a consideration of the stochastic models [11].

A stochastic model was popularized amongst haematologists by papers such as the one by Enver titled *"Do stem cells play dice?" *[12]. According to this view, it is stochastic events that trigger diverse intracellular programs to regulate the differentiation of cells. The stochastic model recognizes growth factors as the important but permissive regulators of differentiation that support the survival and proliferation of one or a few already determined cell type(s) [13]. Neurobiologists had started acknowledging a long time ago that stochastic events might play an important role during differentiation [14]. In time, however, deterministic models became more popular amongst them [15-17].

Another popular model of differentiation is the continuum model. This model refers to the phenotypical changes that occur in stem cells during the cell cycle [18]. The continuum model is based on the assumption that periodical and fluctuating phenotypical changes in stem cells cause the inevitable heterogeneity of their population, which – in turn, is responsible for the difference in their response to the environment.

However, recognizing the very different nature of events that determines the fate of progenitors or stem cells, both – stochastic and continuum, models have identified intracellular events as having the limiting influence on extracellular factors.

The literature data showing that developmental options of very restricted precursors (lymphoblasts, glial restricted precursors), can be analysed as inclined (predisposed) by both extracellular and intracellular events [19,20], empowered us to transfer similar experimental attempt to investigate differentiation of neural progenitor cells (NHA).

We report here that the simultaneous consideration of more than one differentiation model may help in (a) controlling (understanding) human neural progenitor cells (NHA) differentiation, and (b) recognizing the limitations of typical biotechnological manipulations of cell cultures.

## Methods

### Expansion of GFAP-positive neural progenitors (NHA)

NHA isolated from the cerebrum of human foetuses were purchased from Cambrex (CC-2565, NHA-Normal Human Astrocytes; Walkersville, MD). NHA cells were cultured by plating at a density of 10 000–15 000 cells/cm^2 ^in expansion medium (Cambrex AGM Bullet Kit: 3% FBS – fetal bovine serum, rhEGF – recombinant human epidermal growth factor, insulin and ascorbic acid), in accordance with the manufacturer's protocol. The cells were incubated at 37°C with 5% humidified CO_2 _and allowed to reach confluence during 1–2 days. Once they have reached about 95% or 50% of confluence, the cells were then either used for differentiation purposes or were harvested with 0.25% trypsin and 1 mM EDTA for 5 min at 37°C counted, and passaged.

### Protocols of differentiation

#### 1. Differentiation of GFAP+ neural progenitors to non-neural cells

GFAP-positive neural progenitors (NHA) were plated on six well dishes or uncoated chambers (Nunc) at a density of 10 000–15 000 cells/cm^2 ^and passaged in expansion medium containing 3% FBS, rhEGF, insulin and ascorbic acid (Cambrex AGM Bullet Kit). After every passage, the cells were cultured for 5 days before the next passage was performed.

#### 2. Differentiation of GFAP+ neural progenitors to neuronal and glial populations

GFAP-positive neural progenitors (NHA) at the passage 0 were plated at a density of 10 000–15 000 cells/cm^2 ^on Matrigel thin-coated (according manufacturer's protocol, BD Biosciences), chamber slides and cultured in expansion medium (Cambrex AGM Bullet Kit) until reaching confluence. After reaching confluence, the medium was changed to N2- or B27-supplemented DMEM F12 (Invitrogen). A final concentration of 10 ng/ml of growth factor was achieved with the optional addition of either bFGF – basic fibroblast growth factor, SHH – sonic hedgehog or GDNF – glial derived neutrophic factor (Invitrogen). The cells were then cultured under differentiation conditions for 5–7 days, with medium replacement every 2 days.

#### 3. Differentiation of GFAP+ progenitors to neuronal, glial and non-neural cells

GFAP+ neural progenitors were plated on six well dishes or uncoated chambers at a density of 10 000–15 000 cells/cm^2 ^and kept for one to two passages in expansion medium (Cambrex AGM Bullet Kit). After every passage, the cells were kept for 5 days before the next passage was performed. After reaching confluence, the medium was changed to N2- or B27-supplemented DMEM F12 (Invitrogen). A final concentration of 10 ng/ml of growth factor was achieved with the optional addition of one of the following growth factors: bFGF, SHH or GDNF (Invitrogen). The cells were then cultured under differentiation conditions for 5–7 days, with medium replacement every 2 days.

### Low density seeding

The differentiation protocol 2 and protocol 3 were performed also after seeding of NHA cells passage 0 at the following densities: 7 000, 5 000, 2 500 and 1 000 cells/cm^2^.

After seeding, the cells were cultured in expansion medium (Cambrex AGM Bullet Kit) for 5 days, then the medium was changed to N2- or B27-supplemented DMEM F12 with bFGF (Invitrogen) for 5–7 days; the medium was replaced every 2 days.

### Protocol testing commitment of NHA cells passage 0

Low density seeding used for subcloning purposes was established at 5 000 cells/cm^2^.

NHA cells were plated on Matrigel thin-coated (manufacturer's protocol; BD Biosciences) chamber slides and cultured in expansion medium (Cambrex AGM Bullet Kit) until they reached 50% or 95% confluence. Then the cells were grown for 5–7 days in N2- or B27-supplemented DMEM F12 with bFGF (Invitrogen), with medium replacement every 2 days.

### Immunocytochemistry

Immunocytochemistry assays based on double or triple immunofluorescent labeling were performed. For immunofluorescent studies, the cells were grown on sterile slides or for subcloning experiment in a 16-well immunocytochemistry dishes (Nunc). The cells were fixed in 4% paraformaldehyde in PBS for 15 minutes, permeabilized with 0.1% Triton X-100 for 10 min at room temperature and blocked with 1% donkey serum in PBS for one hour at room temperature. For double or triple immunolabeling, the fixed cells were subsequently incubated with appropriate anti-human primary antibodies (1 h, room temperature) listed in Table 1. Double- or triple-labeling was achieved by simultaneous incubation with a suitable combination of species-specific fluorochrome-conjugated secondary antibodies (1 h, room temperature). For double immunolabeling, the mix of donkey anti-rabbit AlexaFluor^®^488, 1:250 and donkey anti-mouse AlexaFluor^®^594, 1:250 antibodies (Molecular Probes) were applied; for triple-labelling the following combination of antibodies was used: donkey anti-rabbit AlexaFluor^®^488, 1:250, donkey anti-mouse AlexaFluor^®^594, 1:250, donkey anti-goat AlexaFluor^®^350, 1:250; (Molecular Probes). Controls with secondary antibodies alone, and with matched isotype controls in place of primary antibodies were processed in the same manner. After a final rinse with PBS, the slides were mounted using ProLong^® ^Gold Antifade Reagent (Molecular Probes). For nuclei staining, the ProLong^® ^Gold Antifade Reagent with DAPI (Molecular Probes) was used. The slides were coverslipped and examined using an Olympus BX-41 fluorescence microscope.

**Table 1:** Primary antibodies applied for immunocytochemistry purposes

**I Ab**	**Host**	**Manufacturer**	**Dilution**
anti-GFAP	mouse	Chemicon; MAB360	(1:400)
	goat	Santa Cruz Biotechnology, Inc.; sc-6171	(1:50)
anti-CD44	mouse	Santa Cruz Biotechnology, Inc.; sc-7297	(1:100)
anti-MAP2	rabbit	Santa Cruz Biotechnology, Inc.; sc-20172	(1:100)
anti-FIBRONECTIN	rabbit	Sigma; F 3648	(1:200)
anti-VIMENTIN	goat	Chemicon; AB-1620	(1:40)
anti-SOX2	rabbit	Chemicon; AB5603	(1:1000)
Anti-TH	mouse	Santa Cruz Biotechnology, Inc.; sc- 25269	(1:100)
	rabbit	Pel-Freez Biologicals; P40101-0	(1:500)
anti-GABA	rabbit	Chemicon; AB141	(1:50)
anti-β-III-TUBULIN	mouse	Chemicon; MAB1637	(1:100)
anti-NESTIN	mouse	Santa Cruz Biotechnology, Inc.; sc-23927	(1:100)
anti-S100β	mouse	Santa Cruz Biotechnology, Inc.; sc-81709	(1:100)

### Cell Counting

Immunoassayed cells were analyzed with an Olympus BX-41 fluorescence microscope. Ten fields were counted for each cover-slip – with each field containing minimum 50 cells. The number of positive cells was averaged from at least three cover-slips from three independent experiments. The percentages of cells positive for MAP2, GFAP and/or CD44 were calculated after differentiation.

### Real time PCR

The RNA samples were obtained from undifferentiated NHA cells and from non-neural population (differentiation protocol 1), neural cells (differentiation protocol 2) and population consists of both cell types (differentiation protocol 3).

Total cellular RNA was isolated from cell cultures using NucleoSpin RNA/Protein kit (MACHERY-NAGEL) according to the manufacturer's protocol. Contaminating residual genomic DNA was removed by DNase I digestion and an aliquot containing 1 μg of isolated RNA was reverse transcribed into single-stranded cDNA in a final volume of 40 μl using QuantiTect Reverse Transcription Kit (Qiagen).

A Real time quantitative PCR was performed on a Rotor Gene 6000 (Corbett, Life Sciences, Australia) instrument utilizing the SYBR^® ^Green Master Mix (Applied Biosystems, CA). Following MAP2 primers were used for amplification:

F 5' GCACTTCAAGGGAAGCTGAT 3', R 5' ATCAAATGGTCCACTAGGCG 3'.

Each sample was amplified in triplicate (for each run) in a reaction volume of 10 μl containing 50 ng cDNA, 1× SYBR Green Master Mix and forward and reverse primer 35 ng each. The cycling conditions were as follows: 3 min at 95°C (polymerase activation) followed by 40 cycles of 15 s at 95°C (denaturation), 45 s at 59°C (annealing) and 1 min at 72°C (extension). *ACTB *was used as a reference gene for normalization of target genes expression levels. Normalized relative expression level for a given gene of non-neural cells, cell population consisted of neural and non-neural cells and neural population obtained after differentiation versus undifferentiated cells was calculated utilizing the method described previously by Pfaffl et al. and implemented into computer application REST2005 [21], based on each sample's average CT value and each gene's average PCR efficiency. Additionally, the Real time PCR products obtained after 20 cycles were analyzed on 2% agarose gel electrophoresis.

### Western blot analysis

The protein samples were isolated from undifferentiated NHA cells and from non-neural population (differentiation protocol 1), neural cells (differentiation protocol 2) and population consists of both cell types (differentiation protocol 3).

The protein extracts from cell cultures were obtained with the use of NucleoSpin RNA/Protein kit (MACHERY-NAGEL). The commercially available Brain Tissue Lysate was purchased from Abcam and used as positive control in Western blot analysis. Equal amounts of protein extracts, 25 μg per line, were separated by 5% SDS-PAGE followed by transfer onto Immobilon™-P polyvinylidene difluoride membranes (Sigma-Aldrich). After blocking nonspecific binding the membranes were incubated for 1 hour at room temperature with rabbit anti-MAP2 primary antibody (Santa Cruz Biotechnology, Inc.; sc-20172). After extensive washing in TBST buffer (TRIS Buffered Saline with Tween 20; pH 7.6) the membranes were incubated for 1 hour at room temperature with anti-rabbit secondary antibody (Santa Cruz Biotechnology, Inc.), diluted 1:2000. Membrane was then washed in TBST buffer and finally in TBS buffer (TRIS Buffered Saline; pH 7.5); antigen-antibody complexes were detected by enhanced chemiluminescence, using Chemiluminescence Luminol Reagent (Santa Cruz Biotechnology, Inc.) and visualized with the use of BIO-RAD camera and Quantity One software.

### Statistical analysis

The effect of cell density on non-neural population percentage was assessed by the non-linear estimation by fitting the experimental data to exponential curve. Log-linear analysis was employed in order to analyse effects of medium and growth factors used in cell culture to increase percentage of neuronal cells. The differences in gene expression levels were determined by the bootstrap randomization technique, implemented in program REST2005. Statistical significance was assumed for P-value ≤ 0.05.

## Results

### NHA cells at the passage 0 showed one homogenous phenotype

**Figure 1:**
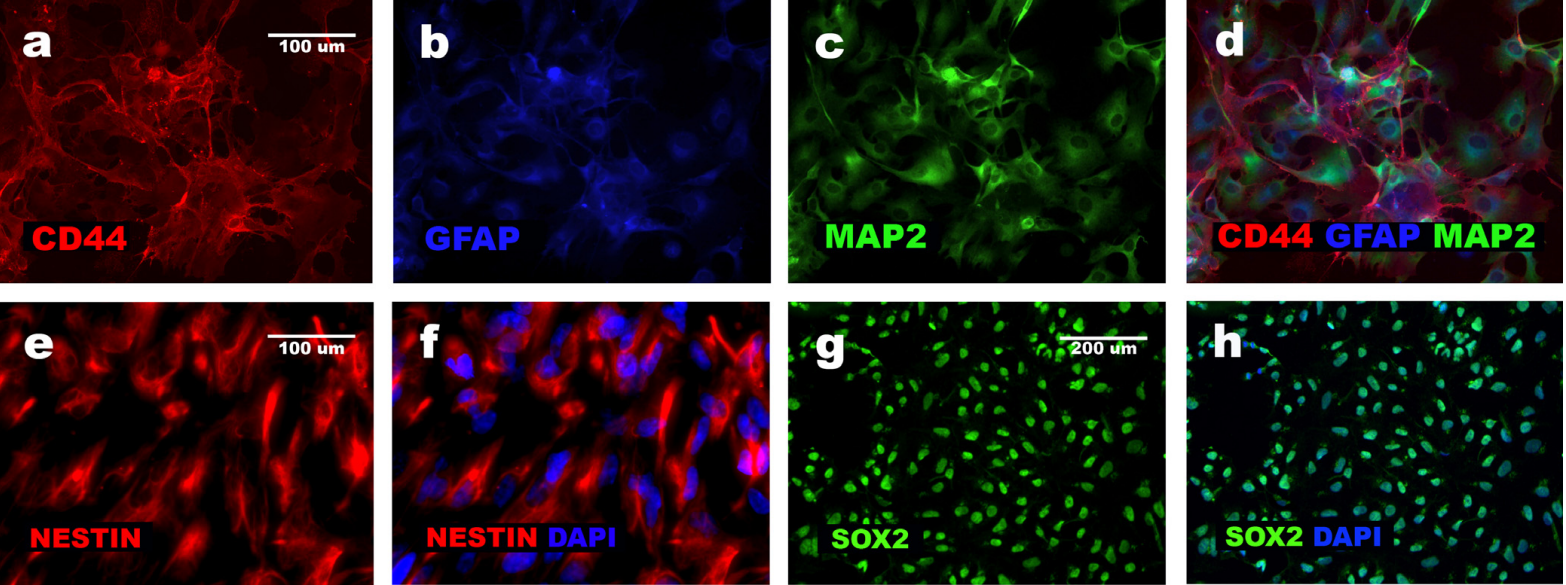
**Expression of neuronal, glial and progenitor markers in undifferentiated NHA cells**. Immunocytochemistry results show high expression of CD44, GFAP and low expression of MAP2 in NHA cells passage 0 (a, b, c, d) and demonstrate presence of NESTIN (e, f) and SOX2 (g, h) in initial uncommitted NHA as neural progenitors.

**Figure 2:**
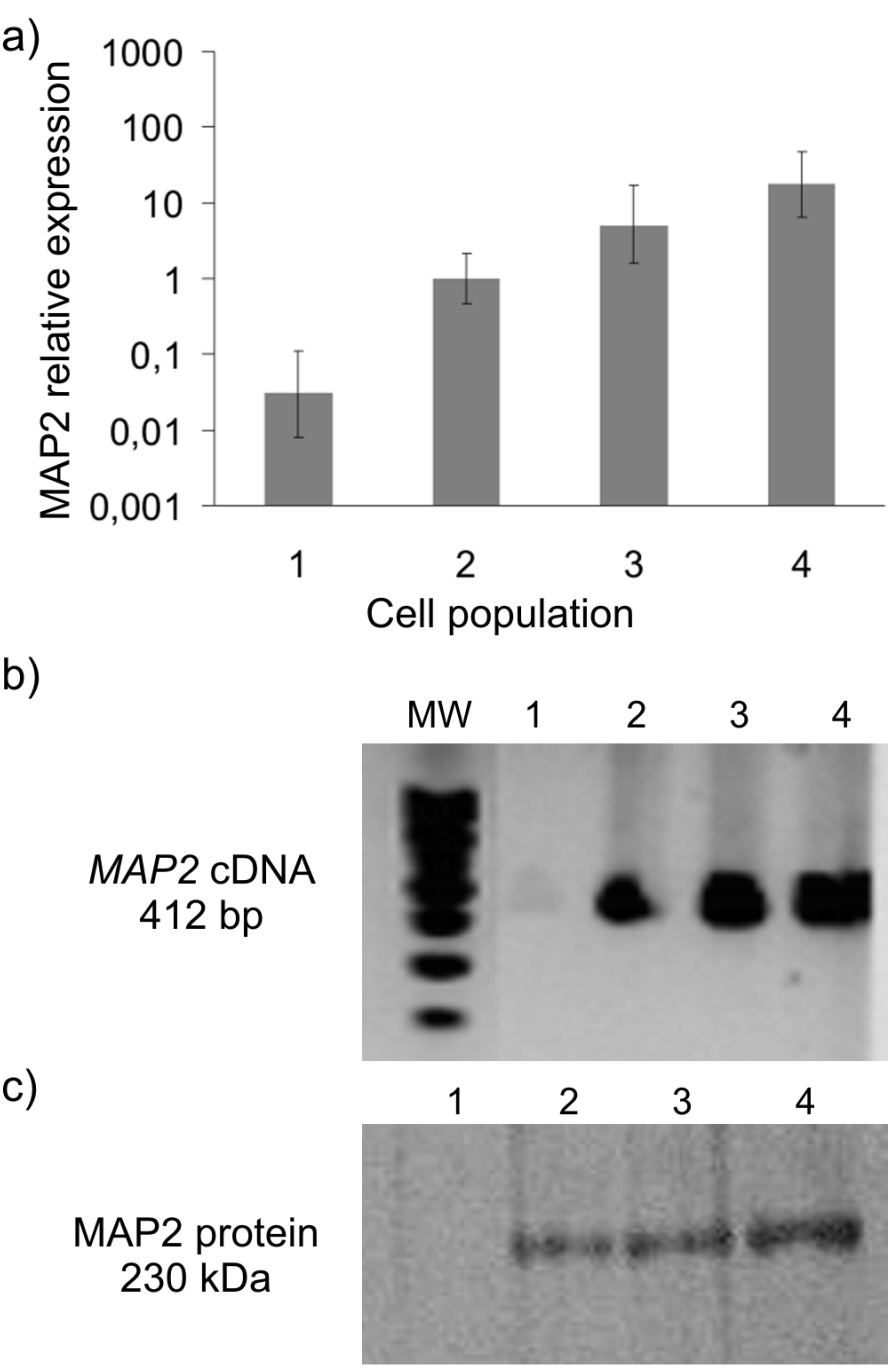
**Expression pattern of *MAP2 *gene during NHA differentiation**. Results of Real time PCR and Western blot analysis confirmed presence of *MAP2 *gene product in undifferentiated NHA cells both at mRNA and protein level and revealed changes in *MAP2 *expression in neural and non-neural population after differentiation. (a) Results of Real time PCR for *MAP2 *relative expression presented as mean of relative expression and respective standard error. (b) Products of Real time PCR for *MAP2 *cDNA visualized on agarose gel electrophoresis. (c) Detection of levels of MAP2 protein expression by Western blot analysis. RNA and protein was isolated from particular cell populations (1, non-neural cells; 2, undifferentiated cells; 3, population consists of neural and non-neural cells; 4, neural cells). MW, molecular weight marker.

We have already shown that – prior to differentiation, proliferative NHA cells express GFAP, β-III-TUBULIN, VIMENTIN, NESTIN, CD44 and MAP2 [1,2]. In this report, we have also shown a co-expression of CD44, GFAP and MAP2 by means of triple staining and SOX2 expression by a single staining (Figure 1). MAP2 expression in original population of NHA cells was confirmed by Western blot and Real time PCR (Figure 2). MAP2 expression was low in NHA undifferentiated cells. Statistical analysis revealed that expression of *MAP2 *gene at mRNA level is downregulated in non-neural cells and upregulated in neural cells and mix of both populations derived according to particular differentiation protocols as compared to initial NHA cells, (P ≤ 0.05); (Figure 2).

### Environmental factors affect the fate of NHA cells

Two completely different derivatives could be obtained following exposure of NHA cells to two diverse environments.

**Figure 3:**
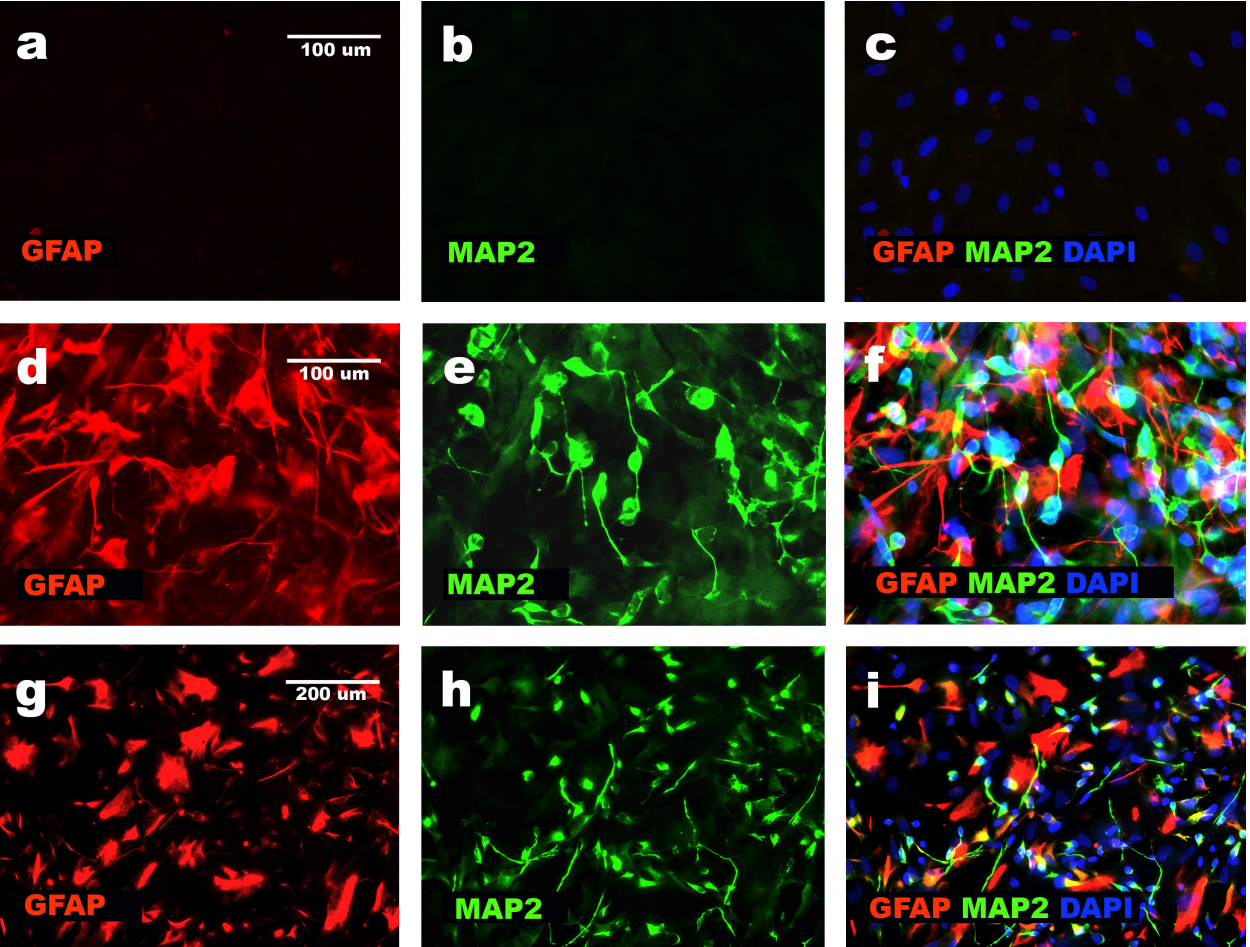
**Neural and non-neural differentiation of NHA cells resulted in changes in MAP2 and GFAP expression**. Immunocytochemistry data based on double staining show: silencing of neural markers [MAP2, GFAP] in non-neural cells obtained according to protocol 1 (a, b, c); presence of equal proportion of neuronal [MAP2+, GFAP-] and glial [GFAP+, MAP2-] cells after differentiation of NHA cells according to protocol 2 (d, e, f) and presence of the third non-neural population of MAP2 and GFAP negative cells accompanying neuronal [MAP2+, GFAP-] and glial [GFAP+, MAP2-] population as a result of protocol 3 (g, h, i).

**Figure 4:**
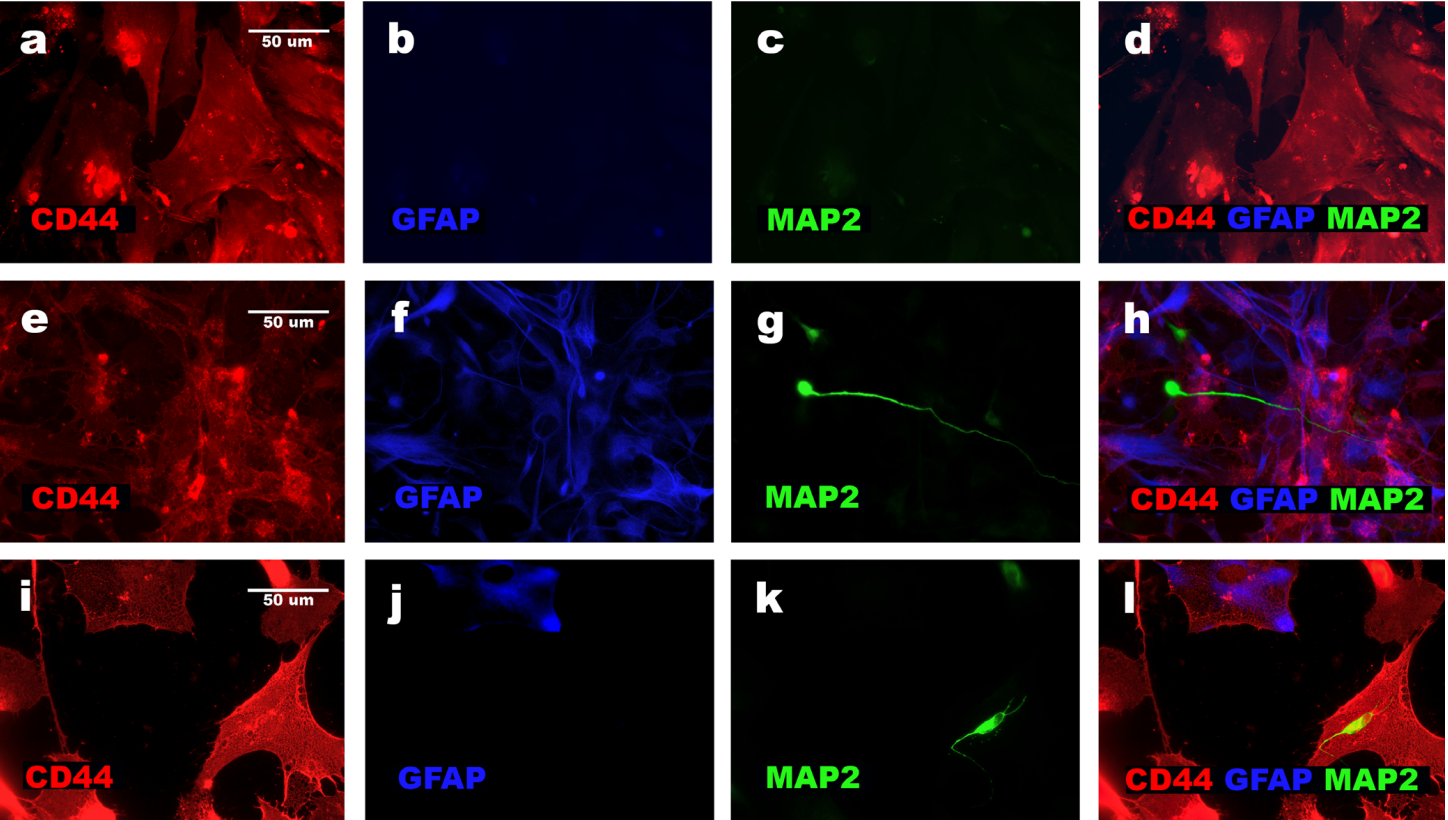
**Three derivatives of NHA cells present distinct expression of MAP2, GFAP and CD44**. Results of triple immunostainings applied to derivatives of NHA cells obtained according particular differentiation protocols reveal: formation of one phenotypically homogenous [CD44+, MAP2-, GFAP-] non-neural population as a result of protocol 1 (a, b, c, d); presence of neuronal [MAP2+, CD44-, GFAP-] and glial [GFAP+, CD44+, MAP2-] populations in case of protocol 2 (e, f, g, h) and appearance of the third population of non-neural cells [CD44+, MAP2-, GFAP-] coexisting with neuronal [MAP2+, CD44-, GFAP-] and glial [GFAP+, CD44+, MAP2-] lineage in case of protocol 3 (i, j, k, l).

Confluent NHA cells passage 0 exposed to N2- or B27-supplemented DMEM F12, differentiated with almost 100% efficiency to neural cells (glial and neuronal) (Figures 3 and 4; Protocol 2). Undifferentiated cells presenting phenotype GFAP+, MAP2+, and CD44- were observed only occasionally after following protocol number 2 (Table 2).

**Table 2:** The proportion of neuronal and glial cells obtained in different culture conditions

**Differentiation condition** NHA-passage 0; 5–7 days	**DMEM F12 with N2**				**DMEM F12 with B27**			
	
	-	**bFGF**	**SHH**	**GDNF**	-	**bFGF**	**SHH**	**GDNF**
**Neuronal cells** MAP2+ GFAP- CD44-	50%	56%	54%	57%	50%	54%	55%	55%
**Glial cells** GFAP+, CD44+, MAP2-	46%	41%	44%	42%	47%	44%	44%	43%
**Undifferentiated cells** MAP2+, GFAP+, CD44-	4%	3%	2%	2%	3%	2%	3%	2%

NHA cells that were seeded at a density of 10 000–15 000/cm^2^, and exposed for one passage to expansion medium (Cambrex medium with 3% FBS, rhEGF, insulin, and ascorbic acid), until they reached confluence, and subsequently exposed to N2- or B27-supplemented DMEM F12, produced three main populations of cells: (1) glial [GFAP+, CD44+, MAP2-], (2) neuronal [MAP2+, CD44-, GFAP-], and (3) non-neural cells robustly producing FIBRONECTIN [CD44+, VIMENTIN+, GFAP-, MAP2-, S100β-, SOX2-] in brief [CD44+, GFAP-, MAP2-]. Only 5–10% of the cells presented phenotype [CD44+, GFAP-, MAP2-] after differentiation at the passage 1 (Figures 3 and 4; Protocol 3). NHA cells at the passage 3 produced approximately 40% of cells with [CD44+, GFAP-, MAP2-] phenotype and equal proportions of neuronal and glial cells (Figures 3 and 4; Protocol 3). Cells with phenotype [GFAP+, MAP2+ CD44-] were – as before (Table 2), rarely observed after following protocol 3.

**Figure 5:**
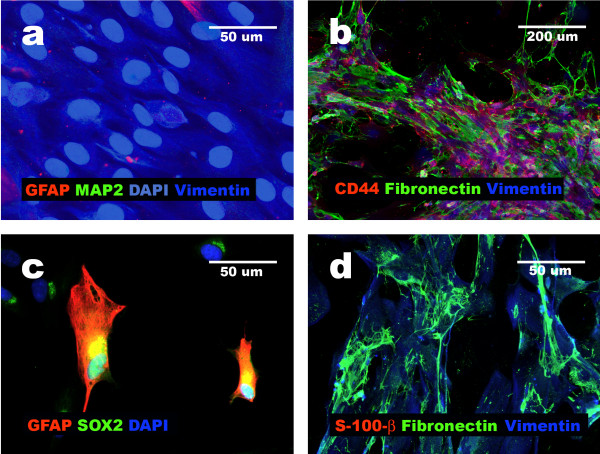
**Characterization of non-neural derivatives of NHA cells cultured in expansion medium**. Immunocytochemistry results demonstrate silencing of neural markers [GFAP, S100β, MAP2] expression, presence of VIMENTIN, CD44 and abundant expression of FIBRONECTIN (a, b, d) as well as lack of SOX2 (c) in NHA cell derivatives obtained after four passages in Cambrex expansion medium with FBS (differentiation protocol 1).

On the other hand, NHA cells passage 0 exposed to the expansion medium for 4–5 passages differentiated – with 100% efficiency, into cells expressing CD44, VIMENTIN, and robustly producing extracellular matrix protein – FIBRONECTIN (Figure 5; Protocol 1). The similar effect was also obtained when NHA cells passage 0 were seeded at a density of 1 000 cells/cm^2 ^(Table 3). These cells expressed no MAP2, GFAP, SOX2 and S100β (Figure 5) as well as NESTIN and β-III-TUBULIN (data not shown) and lost ability to provide neuronal [MAP2+, GFAP-] and glial [GFAP+, MAP2-] cells after exposure to the neural differentiation medium (DMEM F12 N2 with or without growth factors). To this end, we temporarily recognized these cells as non-neural.

**Table 3:** Influence of seeding density on percentage of non-neural cells obtained after differentiation

**Seeding density**	**Non-neural cells percentage**
15 000 cells/cm^2^	0%
10 000 cells/cm^2^	0%
7 000 cells/cm^2^	10%
5 000 cells/cm^2^	20%
2 500 cells/cm^2^	60%
1 000 cells/cm^2^	100%

Changes in density seeding of NHA cells at the passage 0 also influenced the fate of GFAP-positive progenitors presented here (see below).

### Decreasing seeding density of NHA cells at the passage 0 caused appearance of non-neural cells

NHA cells at the passage 0 were seeded at densities of 15 000 cells/cm^2^, 10 000 cells/cm^2^, 7 000 cells/cm^2^, 5 000 cells/cm^2^, 2 500 cells/cm^2 ^and 1 000 cells/cm^2^. In spite of the fact that they were all at the passage 0, the cells that were seeded at densities lower than 7 000 cells/cm^2 ^were observed to present [CD44+, GFAP-, MAP2-, SOX2-] phenotype, once confluence was reached in the Cambrex medium with 3% FBS, rhEGF and ascorbic acid, followed by seven days exposure to DMEMF12 with N2 (B27). Influence of seeding density on NHA cells passage 0 differentiation is presented in Table 3.

In order to evaluate the effect of cell density on percentage of non-neural population, experimental data were fitted to exponential curve y = 122.81 * exp(-0.0004x) (p < 0.0001). Regression analysis explained 90% of variability in percentage of non-neural population (r = -0.09509; R^2 = 0.9043). These results point to the significant influence of the environmental factor (seeding density) on NHA cells differentiation.

### Subcloning of NHA cells do not suggest commitment before serum starvation

**Figure 6:**
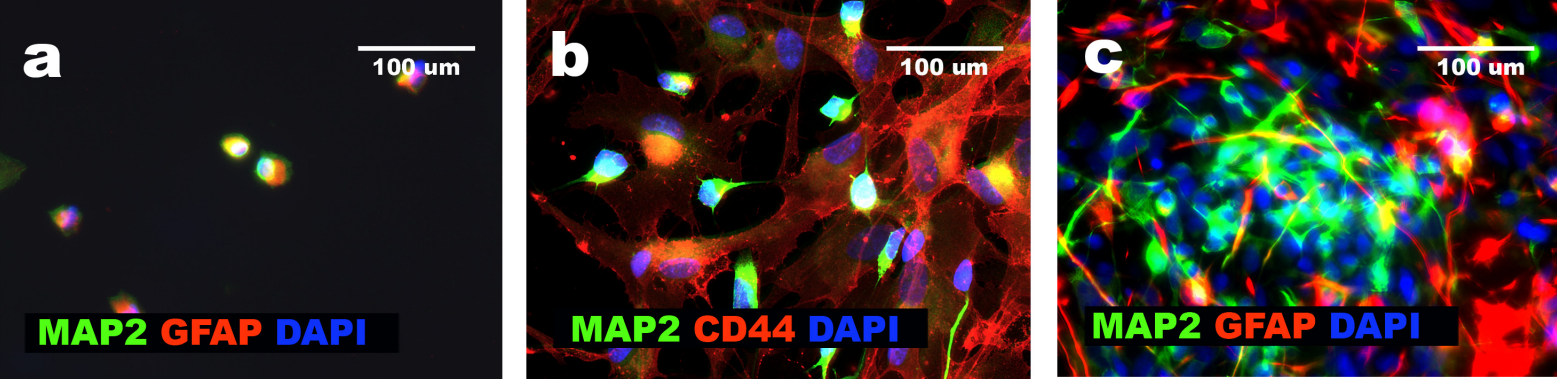
**Subcloning method revealed undifferentiated NHA cells as non-committed homogenous cell population**. Immunocytochemistry results show: co-expression of GFAP and MAP2 in undifferentiated NHA cells passage 0 seeded at a very low density (5 000 cells/cm^2^) (a); equal "non-colonial" distribution of cells representing phenotype [MAP2+, CD44-] and phenotype [CD44+, MAP2-] obtained as a result of neural differentiation of NHA cells which previously reached confluence in medium with serum (b); ability of NHA cells to form colonies obtained as a result of neural differentiation of non-confluent cells (c).

After low density seeding – 5 000 cells/cm^2 ^(Figure 6a), NHA cells passage 0 proliferated in expansion medium and reached confluence. As was described above, confluent NHA cells were subsequently differentiated to neuronal and glial cells when exposed to N2- (or B27-) supplemented DMEM F12. Fifty percent of neuronal [MAP2+, GFAP-] and forty-six percent of glial cells [GFAP+, MAP2-] were obtained. In case when NHA cells reached confluence before serum starvation, equal distributions of two kinds of cells – rather than "colonies" of neuronal and glial cells, were observed in the early stages of differentiation (i.e., 2–3 days of exposure to DMEM F12 with N2 or B27), as well as after 5–7 days of differentiation (Figure 6b).

When colonies of neuronal and glial cells were observed, however, radical change in the differentiation protocol was required to obtain them. Medium with serum had to be replaced with DMEM F12 with N2 (B27) before reaching confluence by the original NHA cell culture (passage 0, seeding density 5 000 cells/cm^2^). Presence of colonies after that modification was a consequence of the fact that committed in DMEM F12 with N2 (B27) at low density stage glial and neuronal cells were proliferating within free of cells spaces forming colonies (Figure 6c). It proved that committed, proliferating cells forming colonies could be detected by means of presented here subcloning method.

### GDNF, SHH and bFGF did not radically change the ratio between neuronal and glial cells

NHA cultured in DMEM F12 with N2 or B27 generated neuronal and glial cells. After 5–7 days of differentiation, we observed cell populations of 50% neuronal and 46% glial and approximately 4% of undifferentiated cells [GFAP+, MAP2+, CD44-] (Table 2). However, with the addition of either bFGF, GDNF, or SHH, the populations turned out to be 54–57% neuronal and 41–44% glial (Table 2).

Statistical analysis indicated that influence of growth factors and supplements used in cell culture to increase percentage of neuronal cells was not significant; (P = 1.00).

### Presence of GABA and TH

**Figure 7:**
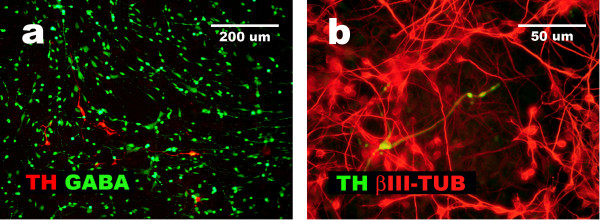
**Differentiation of neuronal lineage of NHA cells resulted in presence of GABA-ergic and catecholaminergic cells**. Immunocytochemistry data show presence of GABA-positive and TH-positive cells as derivatives of neural differentiation of NHA cells according to protocol 2 (a, b) and demonstrate that TH positive cells present β-III-TUBULIN expression (b).

Neuronal MAP2 positive cells obtained after NHA cells differentiation were able to produce GABA (gamma-aminobutyric acid) and express TH (tyrosine hydroxylase). GABA-ergic positive cells dominated (Figure 7).

## Discussion

The main goal of our current research was to obtain the highest possible percentage of biotechnologically useful-cetacholaminergic and GABA-ergic, neuronal cells via differentiation of NHA neural progenitors. To this end, we considered the influence of intrinsic and extrinsic factors during NHA cells differentiation. NHA cells have been described as GFAP-positive neural progenitors in our previous reports [1,2], but here, for the first time, we showed that these cells express SOX2 at the passage 0. In spite of the fact, that expression of SOX genes are associated with neural stem cells and SOX proteins are regarded as markers of neural primordium [22], NHA differentiation status is not obvious. According to our results these cells were identified as neural progenitors, but we couldn't exclude a possibility that NHA may reveal features of stem cells [1,2]. Anyway, the influence of intrinsic and extrinsic events should be considered not only in the context of stem cells, since also progenitors are biotechnologically valuable.

We found nothing to suggest that NHA cells culture at the passage 0 consists of two or more cell populations presenting permanent, diverse intrinsic regulators of differentiation. In other words, we found no data to support the idea that the fate of NHA cells is determined at the passage 0 before serum starvation (Figures 1 and 6) [1,2]. However, we cannot exclude the possibility that NHA cells were changing their phenotype periodically and discretely in accordance with the continuum model [18]. A hypothesis of the permanent fate pre-determination was also undermined by the results showing that uncommitted NHA cells were very selectively differentiated in serum-free conditions into neural cells. On the other hand, in medium containing serum, NHA cells were directed, after 4–5 passages or extremely low density seeding at the passage 0, with 100% efficiency towards cells recognized temporarily as non-neural on the basis of presented phenotype [CD44+, VIMENTIN+, FIBRONECTIN+, SOX2-, MAP2-, GFAP-, S100β-, NESTIN-, β-III-TUBULIN-] and disability to differentiate into the neuronal derivatives (Figure 5; for NESTIN and β-III-TUBULIN data not shown). Provided evidences entitled to regard this population as non-neural, nevertheless, defining exact phenotype of these cells requires additional investigation.

Our results showed that environmental changes triggered two different types of differentiation: neural and non-neural (Figures 3, 4 and 5). These data fit the instructive model of differentiation. It also showed, that time of exposure to a particular environment is important during the differentiation of NHA progenitors. The role of the duration of exposure to a particular environment in regulating differentiation has been recognized as a kinetic factor [23].

In spite of findings on the determination of neural versus non-neural fate by means of simple environmental manipulation, we were unable to obtain strictly neuronal or strictly glial cells from any of the cell culture conditions tested. This shows the inadequacy of the instructive model in explaining how glial *vs*. neuronal fate determination is regulated during GFAP positive neural progenitors (NHA) differentiation. We should ask "why" initially uncommitted NHA cells always end up differentiating into two types of neural cells – i.e., neuronal and glial, even when exposed to different – serum free environments. Stochastic and continuum models of differentiation do offer the answer to this question. In accordance with the stochastic model, stochastic events can trigger intracellular developmental diversity and cause the appearance of two (or more) different responses to one environment [11]. Referring to the Enver suggestion that "hematopoietic stem cells play dice" [12], we may suggest that after serum starvation "NHA neural progenitors toss a coin and – getting a head, choose a neuronal fate; whereas getting tail meant choosing a glial fate." The continuum model – with its fluctuating reversible changes during cell cycle temporarily biasing the fate of progenitors, also fits the results that we observed. The stochastic or continuum nature of the process regulating neuronal *vs*. glial cells determination was supported by the results of our sub-cloning experiment (Figure 6). In essence, these models can explain our data, which shows that growth factors such as bFGF, GDNF, or SHH were not able to increase dramatically the percentage of neuronal cells within, five to seven days exposure to the serum free medium (Table 2). Both models are based on the thesis that intracellular events cause certain cellular independence in terms of their responses to environmental changes. In our experiments, growth factors were unable to trigger only neuronal differentiation from neural progenitors (NHA), which is in accordance with stochastic and continuum models.

It seems important, from the biotechnological point of view, that we were able to limit the percentage of non-neural cells [CD44+, VIMENTIN+, FIBRONECTIN+, GFAP-, MAP2-, S100β-, SOX2-] (Figures 3 and 4) since cells robustly producing FIBRONECTIN could be hazardous as an element of graft in the central nervous system [24,25]. Moreover these cells were unable to provide neuronal cells and therefore we recognized them as useless considering our project target – the procurement of neuronal cells. In spite of the successful elimination of non-neural cells, we did not manage to increase significantly the ratio between neuronal *versus *glial cells (Table 2). It could be disappointing that we were almost unable to break the symmetrical proportion between neuronal and glial cells while testing several growth factors. Nevertheless, we can still propose an answer for the question of why either stochastic or continuum models, basing on the primary role of intrinsic factors and the permissive role of extrinsic factors, can describe the biologically designed process of neural progenitors (NHA) differentiation. It is very likely that *in vitro *glial cells – as in *in vivo *ones, support neuronal cell growth, differentiation, and maturation [26,27]. Since the biological process regulating the differentiation of neural progenitors seems to be rational in its simultaneous provision of glial and neuronal cells, biotechnological manipulations leading to the obtainment of only – or almost only, neuronal cells could result in the elimination of natural supporters of neuronal cells.

## Conclusion

**Figure 8:**
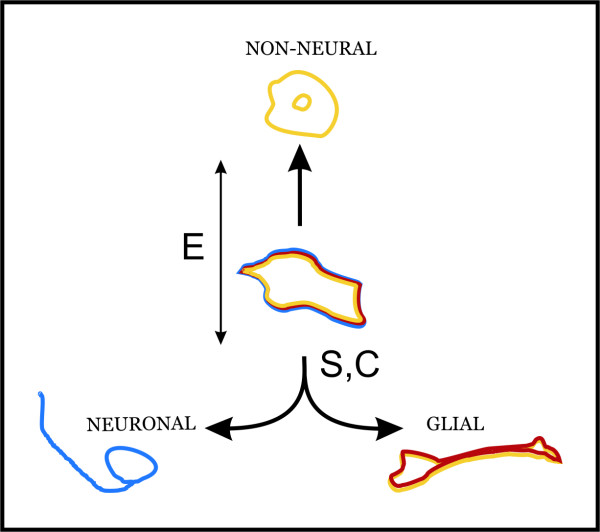
**Scheme presenting differentiation ability of NHA cells according to deterministic, stochastic and continuum model**. In accordance with the deterministic model, neural or non-neural differentiation of NHA cells is a result of different environmental conditions (E), whereas unbreakable balance between neuronal and glial derivatives seems to be consistent with both stochastic (S) and continuum models (C).

We made two key observations from our experiments. The first is that environmentally regulated bifurcation towards neural *vs*. non-neural cells occurred in accordance with the deterministic model. The second is that environmentally unbreakable, symmetrical, and simultaneous differentiation towards glial and neuronal cells occurred in accordance with the stochastic and continuum models (Figure 8). However, we cannot determine whether the stochastic or the continuum model is better in describing neural differentiation of NHA progenitors. Nevertheless, approving either one of those two models means accepting the limitations of commonly used cell cultures manipulations to increase percentage yield of neuronal cells. Our approach allowed for the elimination of non-neural cells, and the maximal procurement of about 60% neuronal cells. In the application of more manipulative biotechnological procedures, biological reasons for the limitations of typical methods should be carefully and meticulously scrutinised, since it is possible that physiological cooperation between neuronal and glial cells lays hidden behind these limitations. Still, we were able to show that taking into account more than one model of differentiation can be helpful during biotechnological preparation of cells useful for transplantology purposes.

## Authors' contributions

MW and PR designed project. PR, KH–B, SP performed cells cultures. MW, MZ participated in obtaining immunocytochemistry data. PR was responsible for supervising and founding acquisition. PPL, BK, SMG and ASA participated in experiments required for revision (immunocytochemistry, western blot and real time PCR) and in revision preparation. SMG performed statistical analysis. All authors participated in analysis and interpretation of obtained data. All authors have been involved in drafting the manuscript. All authors have given approval of the final version of the manuscript.
